# Diaphragmatic Ultrasonography in Preterm Newborns With Respiratory Distress Syndrome on Invasive Mechanical Ventilation: An Observational Study

**DOI:** 10.1111/jpc.70102

**Published:** 2025-06-03

**Authors:** Cleuma Oliveira Soares, Caroline Almeida Campbell, Mariana Souza Azevedo Moura, Rodrigo Santiago Barbosa Rocha

**Affiliations:** ^1^ Universidade Do Estado Do Pará – UEPA Belém Pará Brazil; ^2^ Fundação Santa Casa de Misericórdia Do Pará Brazil; ^3^ Fundação Santa Casa de Misericórdia Do Pará Belém Pará Brazil; ^4^ Programa de Pós‐graduação Em Reabilitação e Desempenho Funcional Universidade Do Estado Do Pará – UEPA Brazil

**Keywords:** diaphragm, mechanical ventilation, preterm newborn, ultrasonography

## Abstract

**Aim:**

In preterm newborns, Respiratory Distress Syndrome (RDS) is one of the leading causes of mortality. Treatment often requires invasive mechanical ventilation (IMV), which can cause diaphragmatic dysfunction. Ultrasonography is a promising tool for assessing diaphragmatic function in neonates. This study analyses changes in the diaphragm muscle of preterm newborns with RDS undergoing IMV.

**Methods:**

A longitudinal study was conducted with preterm newborns diagnosed with RDS and subjected to IMV. Clinical data were collected, and ultrasonographic evaluations of diaphragm thickness and diaphragmatic thickening fraction (DTF) were performed at two time points: within the first 48 h of life and up to 48 h after extubation.

**Results:**

There was a significant reduction in diaphragmatic thickness (*p* < 0.0001), DTF index (*p* = 0.01), and diaphragmatic excursion (*p* = 0.02) during the intubation period. A moderate correlation was observed between DTF and IMV duration (*R* = 0.62), hospitalisation time (*R* = 0.52), and the risk of reintubation (*R* = 0.55). A moderate correlation was also identified between IMV duration and hospitalisation time (*R* = 0.64) and between diaphragmatic thickness and gestational age (*R* = 0.58). However, the correlation between DTF and gestational age was weak (*R* = 0.48).

**Conclusion:**

IMV leads to diaphragmatic deterioration with increased muscle atrophy in preterm newborns with respiratory distress syndrome.

**Summary**

- What is already known on this topic
  ○ The scientific literature shows that gestational age and low birth weight are factors that contribute to increased hospitalisation time and diaphragmatic dysfunction.
  ○ Prolonged time on invasive mechanical ventilation leads to diaphragmatic dysfunction and muscle atrophy.
  ○ Ultrasound is an important tool in monitoring diaphragmatic function and can be used as an instrument for extubation from invasive mechanical ventilation.
- What this paper adds
  ○ This study provides updated clinical and demographic data based on a consistent sample of preterm newborns with respiratory distress syndrome treated in a region of the Brazilian Amazon.
  ○ The study highlights that the decline in diaphragmatic trophism and function contributes to prolonged invasive mechanical ventilation and extended hospitalisation time.
  ○ The study highlights that DTF and DE are variables that can be used as indicators for extubation in premature newborns with respiratory distress syndrome.

## 1 Introduction

Newborns exhibit anatomical and physiological differences in their respiratory systems compared to adults. For instance, the diaphragm has a lower proportion of fatigue‐resistant muscle fibres (type I), reduced oxidative capacity, and a smaller total muscle area, making it more prone to muscle fatigue [1].

Additionally, one of the main causes of respiratory failure in PTNBs is Respiratory Distress Syndrome (RDS), also known as hyaline membrane disease. This condition is among the leading causes of high mortality rates in this age group. RDS occurs in preterm newborns due to pulmonary immaturity, which results in insufficient production of pulmonary surfactant. This deficiency leads to altered lung compliance, reduced functional residual capacity (FRC), and progressive hypoxia, significantly increasing the risk of respiratory failure [2].

The treatment of RDS and other conditions related to pulmonary immaturity often requires invasive mechanical ventilation (IMV) or non‐invasive mechanical ventilation. While essential in managing pulmonary diseases in newborns (NBs), IMV can cause complications, such as lung injuries and inflammation [2, 3, 4], accompanied by the development of diaphragmatic muscle weakness and contractile dysfunction—a phenomenon known as ventilator‐induced diaphragmatic dysfunction [5].

The diaphragm muscle architecture of newborns exhibits significant differences in terms of diaphragm thickness and diaphragm excursion when comparing preterm and term infants [6]. This fact may contribute to an increased duration of mechanical ventilation and hospitalisation, but this remains uncertain [7]. A avaliação do diafragma com a ultrassonografia na unidade de terapia intensiva neonatal tem ganhado importância, sendo os parâmetros de espessura diafragmática, fração de espessamento diafragmático e a excursão diafragmática, sendo parâmetros importantes para monitoração e desmame da ventilação mecânica invasiva [8].

Monitoring the diaphragm muscle has gained relevance, with ultrasonography emerging as a method with high sensitivity and specificity for assessment [9, 10]. While extensively studied in the adult population under various conditions, research on its use in neonates remains limited [11, 12]. Therefore, the primary objective of this study is to analyse changes in the diaphragm muscle of preterm newborns with RDS undergoing IMV.

## 2 Methodology

### 2.1 Ethical Aspects

This cross‐sectional observational study was conducted in a Neonatal Intensive Care Unit (NICU), a reference center in the northern region of Brazil, between June and September 2024. The study was approved by the Ethics Committee of Fundação Santa Casa de Misericórdia do Pará under protocol number 6.804.607.

### 2.2 Participants

The study participants were preterm infants born with a gestational age (GA) greater than 27 weeks and less than 36 weeks, diagnosed with RDS and subjected to IMV either at birth or within the first 24 h of life.

Newborns with Acute RDS who did not require IMV or those diagnosed with transient tachypnea of the newborn, pneumonia, pulmonary hypertension, meconium aspiration syndrome, congenital heart defects, pulmonary malformations, postoperative conditions, neonatal anoxia, severe congenital anomalies, periventricular leukomalacia, or seizures were excluded from the study.

The sample size for the study was calculated using the GraphPad StatMate software, version 1.01, with a 5% significance level and 80% test power. The expected sample size for the collection period was the inclusion of 40 patients in the study.

### 2.3 Data Collection Procedure

For data collection, five steps were followed: analysis of medical records and patient selection of patients; signing of the Free and Informed Consent Form (FICF) by the guardians; data collection; diaphragmatic ultrasonography performed within the first 48 h of life and admission, with all patients on invasive mechanical ventilation; diaphragmatic ultrasonography performed up to 48 h after extubation.

Anthropometric data (gestational age, type of delivery, sex, and birth weight) and clinical data during hospitalisation (Apgar scores at 1 and 5 min, surfactant administration, duration of invasive mechanical ventilation, non‐invasive ventilation, oxygen therapy, and length of NICU stay) were collected.

For the ultrasonography procedure, the newborns were positioned in a supine position with their heads in the midline, inside a heated incubator. The GE LOGIQ P6 ultrasound device with a linear transducer of up to 11 MHz was used to evaluate diaphragm thickness. All tests were conducted 1 h after feeding, with monitoring of vital signs. The evaluations were always performed by a trained professional experienced in operating the equipment, with an assistant providing containment and comfort to the participants.

To measure diaphragm thickness, the transducer was positioned on the right mid‐axillary line in the diaphragm's zone of apposition, between the 8th and 11th intercostal spaces. The same anatomical location was used for all participants, and measurements were conducted by the same evaluator. Values from three consecutive respiratory cycles were recorded. The right hemidiaphragm was chosen for all participants due to better feasibility and reproducibility. Thickening values are reported in centimetres [13, 14].

B‐mode ultrasound was used to display the diaphragm image, visualised as a three‐layer structure: a central non‐echogenic layer and two echogenic layers (peritoneum and pleura). Subsequently, M‐mode ultrasound was utilised, and the image was frozen. After selecting the best image, precise measurements of thickness throughout a respiratory cycle were performed.

To determine diaphragm thickness (Tdi), three measurements of muscle thickness were taken. Tdi was measured during end‐expiration (Tdi exp) and end‐inspiration (Tdi insp), and the simple average was calculated. The diaphragmatic thickening fraction (DTF) was calculated using the formula:

(DTF) pela fórmula: (Tdi insp – Tdi exp./Tdi exp) × 100% [13, 14].

After collecting diaphragm thickness and DTF variables, the data were tabulated in a Microsoft Excel spreadsheet and transferred to a blinded researcher who was unaware of the timing of the assessments, ensuring blinding.

## 3 Statistical Analysis

Data analysis was performed using BioEstat 5.2 software. The Shapiro–Wilk test was used to verify the normality of data distribution. Based on data normality, an intragroup significance analysis was performed using ANOVA with Tukey's post hoc test. For correlation analysis between autonomic modulation and the weaning success index, Pearson's correlation test was used for parametric variables. The following correlation thresholds were applied: 0.7–0.9 (positive or negative): strong correlation; 0.5–0.7 (positive or negative): moderate correlation; 0.3–0.5 (positive or negative): weak correlation; 0–0.3 (positive or negative): negligible correlation. Values of *p* < 0.05 were considered statistically significant.

## 4 Results

**FIGURE 1:**
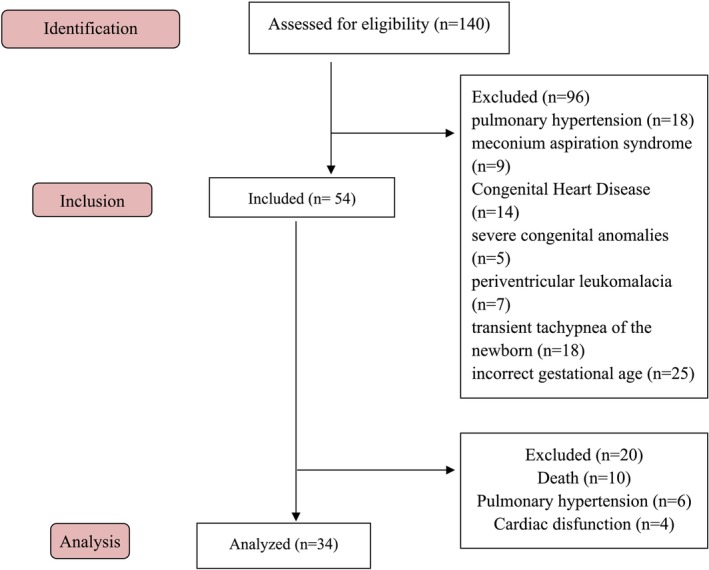
Flowchart of volunteer participation in the study.

The study included 34 preterm newborns with RDS who required IMV (Figure 1). The predominant anthropometric and clinical data (Table 1) of the participants showed a mean GA of 29.3 weeks, categorised as very preterm infants. The majority were female (57.14%) and delivered via caesarean section (71.43%). The average birth weight was 1170 g (low birth weight), and the mean Apgar score was 4.64 at the first minute of life and 7 at the fifth minute. Most participants were classified as small for GA (57.14%). All participants received surfactant therapy. The mean duration (in days) of IMV was 18.3, non‐invasive ventilation (NIV) was 3.36, oxygen therapy was 11.71, and the average length of hospital stay was 53.2 days.

**TABLE 1:** Sociodemographic and clinical data of the study participants.

Variable	Mean ± SD (*n* = 34)	%
Gestational age	29.3 ± 3.53	
Gender		
Male	16	42.86%
Female	18	57.14%
Weight	1170 ± 356.79	
Apgar score		
1 min	4.64 ± 2.47	
5 min	7 ± 2.32	
Vaginal birth	14	28.57%
Caesarean birth	20	71.43%
Small for gestational age	18	57.14%
Appropriate for gestational age	16	42.86%
Surfactant	34	100%
Duration of invasive mechanical ventilation	18.3 ± 19.7	
Duration of oxygen therapy	11.71 ± 10.64	
Length of hospital stay	53.27 ± 27.84	
Reintubation	8	33.33%
Death	4	14.29%

Abbreviation: IMV, invasive mechanical ventilation.

Table 2 presents the data collected regarding IMV at the time of the initial and final assessments. All patients were on invasive mechanical ventilation, with the TCPL mode being the most frequently used.

**TABLE 2:** Parameters of invasive mechanical ventilation.

Variable	Mean ± SD (*n* = 34)	%
Initial parameters of IMV		
TCPL+AC	20	58.82
PCV	14	41.18
Inspiratory pressure (cmH2O)	16 ± 2.1	
Expiratory pressure (cmH2O)	6 ± 0.7	
Fraction of inspiratory oxygen (%)	55.5 ± 7.35	
Final parameters of IMV		
TCPL	34	100
Inspiratory pressure (cmH2O)	13 ± 2.5	
Expiratory pressure (cmH2O)	5 ± 0.8	
Fraction of inspiratory oxygen (%)	32.5 ± 2.5	
Duration of non‐invasive mechanical ventilation	3.36 ± 3.75	

**FIGURE 2:**
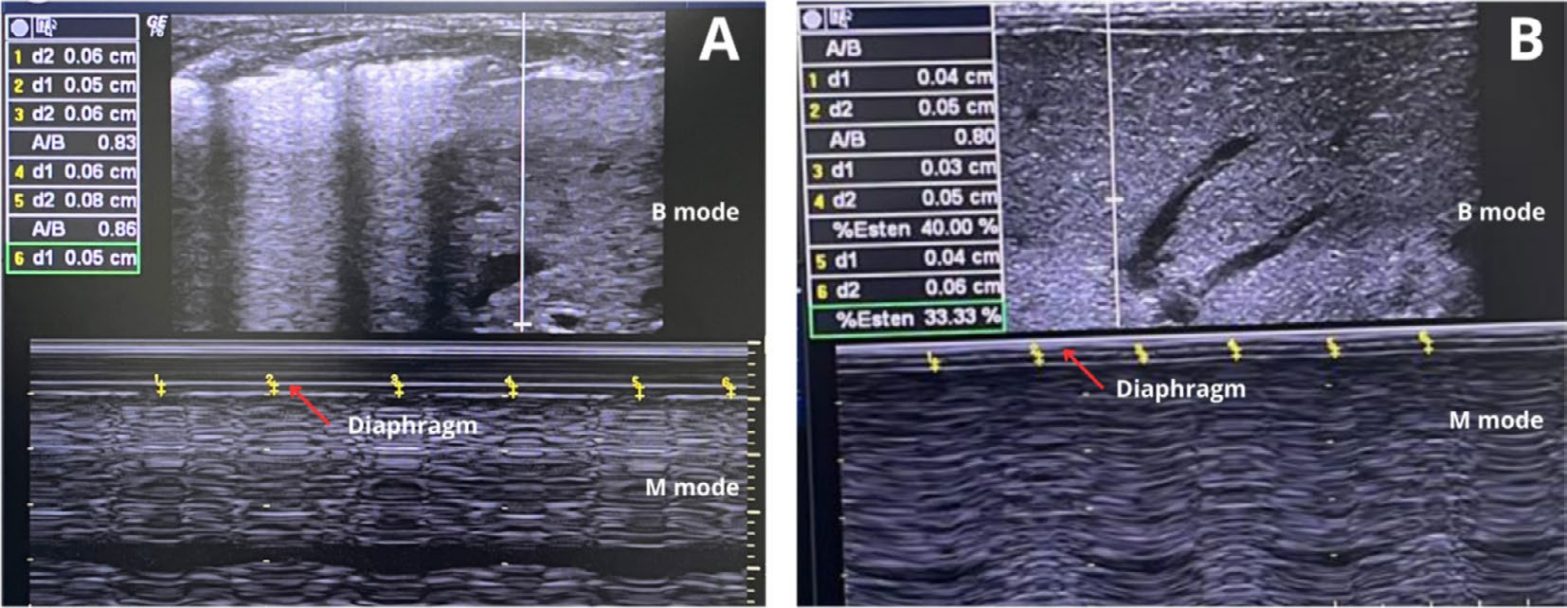
Assessment of diaphragmatic thickness and DTF. (A) Diaphragmatic thickness within the first 48 h of invasive mechanical ventilation. (B) Diaphragmatic thickness within the first 48 h after invasive mechanical ventilation.

**FIGURE 3:**
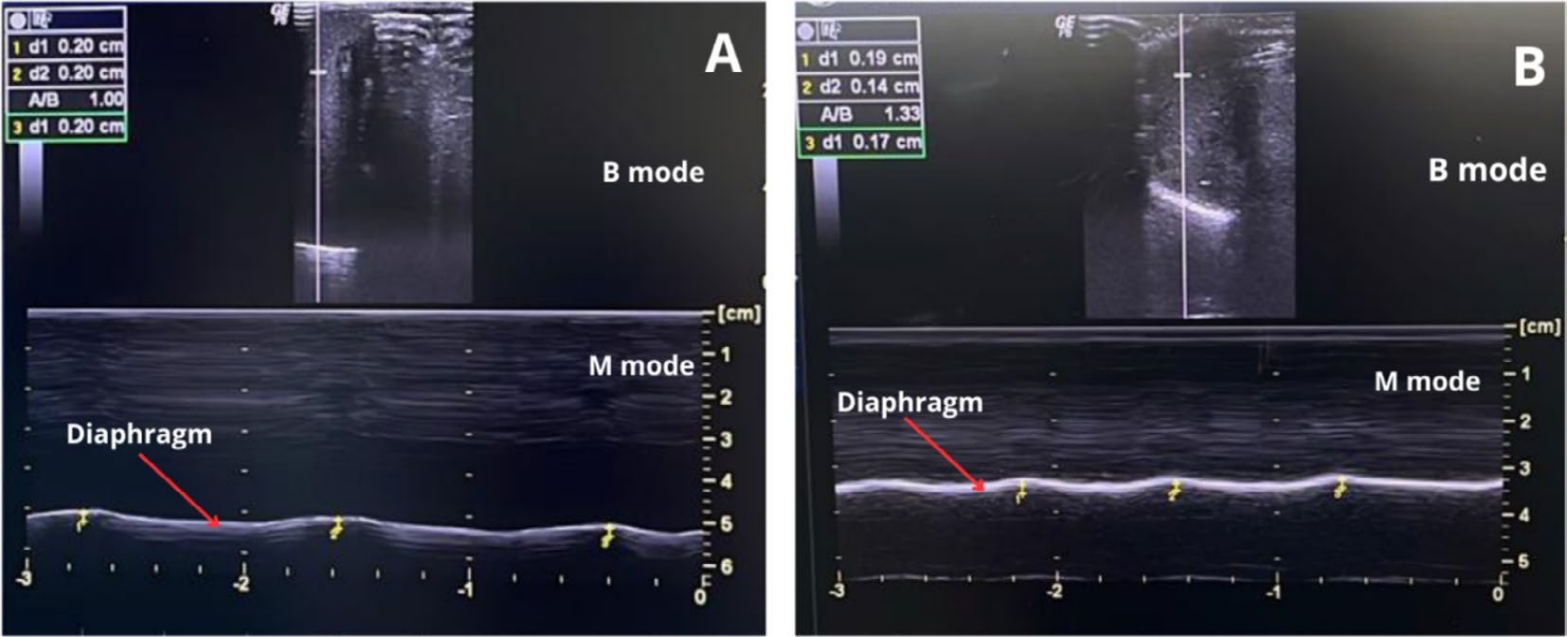
Assessment of diaphragmatic excursion. (A) Diaphragmatic excursion within the first 48 h of invasive mechanical ventilation. (B) Diaphragmatic excursion within the first 48 h after invasive mechanical ventilation.

Table 3 shows the mean and standard deviation (SD) of the initial values, up to 48 h after intubation, and final values up to 48 h after extubation (Table 3). A significant difference was observed in diaphragmatic thickness (*p* < 0.0001), DTF (*p* = 0.01) (Figure 2), and diaphragmatic excursion (*p* = 0.02) (Figure 3) during the period of orotracheal intubation. A significant difference was observed between baseline and final values for diaphragmatic thickness (77.58), DTF (64.46), and DE (70.96).

**TABLE 3:** Baseline and final differences in diaphragmatic thickness and DTF.

Variable	Baseline (Mean ± SD)	Final (Mean ± SD)	Baseline/final difference (%)	*p*
Diaphragmatic thickness (cm)	0.058 ± 0.01	0.045 ± 0.01	77.58	< 0.0001*
DTF (%)	32.00 ± 5.34	20.63 ± 3.26	64.46	0.01*
DE (cm)	0.31 ± 0.16	0.22 ± 0.14	70.96	0.02

Abbreviations: DE, diaphragmatic excursion; DTF, diaphragm thickening fraction.

* *p* < 0.05.

According to the Pearson correlation coefficient, the relationship between DTF and the duration of IMV (*R* = 0.62), length of stay (*R* = 0.52), and reintubation (*R* = 0.55) showed a moderate correlation. Similarly, the duration of IMV and length of stay (*R* = 0.64) also showed a moderate correlation. Diaphragmatic thickness in relation to GA (*R* = 0.58) also showed a moderate correlation, while the correlation between DTF and GA (*R* = 0.48) exhibited a weak correlation (Table 4).

**TABLE 4:** Evaluation of diaphragmatic thickness and thickening fraction, and the correlation between the variables.

Variável	*R*	*p*
Diaphragmatic thickness vs. DTF	0.48	0.05
Diaphragmatic thickness vs. DE	0.51	0.05
DTF vs. DE	0.55	0.05
Diaphragmatic thickness vs. duration of IMV	−0.37	0.14
DTF vs. duration of IMV	0.62	0.04*
DE vs. duration of IMV	0.50	0.05
Diaphragmatic thickness vs. length of stay	−0.31	0.24
DTF vs. length of stay	0.52	0.05*
DE vs. length of stay	0.50	0.05
Diaphragmatic thickness vs. gestational age	0.58	0.04*
DTF vs. gestational age	0.48	0.05
DE vs. gestational age	0.51	0.05
Diaphragmatic thickness vs. reintubation	−0.15	0.55
DTF vs. reintubation	0.55	0.05
DE vs. reintubation	0.5	0.05
Duration of IMV vs. length of stay	0.64	0.02*

Abbreviations: DE, diaphragmatic excursion; DTF, diaphragm thickening fraction; IMV, Invasive mechanical ventilation.

* *p* < 0.05.

## 5 Discussion

This study aimed to identify variations in the diaphragm muscle of preterm newborns with RDS undergoing IMV. We observe a reduction in muscle trophism and correlate acquired muscle atrophy with prolonged IMV duration and increased length of stay in the neonatal intensive care unit (NICU).

The main finding of this study was the reduction in diaphragm thickness and DTF during the IMV period. These parameters were associated with longer NICU stays and failed weaning from IMV. Another significant finding was the direct relationship between diaphragm thickness, DTF, and GA.

In this study, a positive correlation was observed between GA and DTF. Andreazza et al. [12] reported no statistically significant difference in diaphragm thickness (Tdi) and DTF between preterm (PT) and term (T) newborns when correlated. However, it is possible to identify that Tdi is greater in term newborns, suggesting a relationship between Tdi and GA.

The study by Alonso‐Ojembarrena et al. [15] shows results similar to those of this research. In their comparison between term newborns (T) and healthy preterm newborns (PT), they demonstrated that both groups have similar DTF values, although diaphragm dimensions are smaller in the PT group. This indicates that, in this population, in cases without respiratory distress syndrome, diaphragms have a similar percentage of shortening and thickening compared to term newborns.

However, in patients with RDS or transient tachypnea of the newborn, diaphragm function may differ between groups due to variations in chest wall compliance or lung tissue stiffness. Nevertheless, there is a lack of studies that evaluate and relate diaphragm muscle assessment via ultrasound, particularly in terms of diaphragm thickness and excursion, to neonatal age [15].

Moreover, birth weight influences diaphragm thickness. Alonso‐Ojembarrena et al. [16], in their study with 37 preterm newborns (PT) born before 32 weeks of GA, identified that birth weight was the only variable related to diaphragm thickness. They found that the higher the birth weight, the greater the diaphragm thickness at birth and during hospitalisation. They also concluded that the number of days on IMV did not influence diaphragm thickness in preterm infants. However, their research could not establish a relationship between the number of days on IMV and diaphragm thickness.

It is essential to observe that neonatal diaphragm thickness is directly related to weight gain during the NICU stay. Significant weight loss decreases the likelihood of adequate diaphragm muscle development and increases the risk of extubation failure [17]. In our study, this variable could not be tracked, but many newborns experience weight loss after birth, which may reduce diaphragm thickness.

Our study demonstrated a reduction in diaphragm thickness during the IMV period and highlighted that DTF influences IMV duration, hospitalisation length, and an increased chance of reintubation. It is known that remaining on IMV for over 48 h can lead to significant diaphragm mass and strength loss. In this study, all newborns remained on IMV for a period exceeding what is described in the literature. The reduction in diaphragm trophism can be explained by the increase in reactive oxygen species, leading to proteolysis of diaphragm muscle fibres exposed to mechanical ventilation. Furthermore, diaphragm activity is directly related to the lung volumes mobilised during each respiratory cycle. As such, diaphragm function is a predictor of respiratory capacity, and muscle atrophy directly interferes with functionality [18].

Nobile et al. [5] corroborated our findings. In their study with 17 preterm participants (mean GA of 27 weeks), they reported a difference in diaphragm thickness during end‐expiration (TDE), from 0.118 cm on the first day of IMV to 0.104 cm on the last day (*p* = 0.092). Diaphragm atrophy was associated with a higher incidence of extubation failure (58.8%).

This association is not surprising, as newborns have a low diaphragm functional reserve. Additionally, factors such as lower gestational age, the presence of RDS, prolonged IMV periods, and higher ventilatory settings—especially high Peak Inspiratory Pressure and elevated Positive End‐Expiratory Pressure—are associated with deterioration in diaphragm function. Similarly, diaphragm dysfunction can lead to difficult weaning and prolonged mechanical ventilation [19].

BAHGATE et al. [10], evaluated diaphragm thickness and excursion to predict successful extubation of preterm newborns from conventional invasive mechanical ventilation. Their study included 43 newborns, with 34 successful and 9 failed extubations. The successful extubation group had significantly higher diaphragm thickness, DTF, and diaphragm excursion compared to those who failed. They concluded that diaphragm excursion is a useful indicator for successful extubation in preterm newborns.

A systematic review with meta‐analysis [20], which included 11 studies and 828 patients, demonstrated that reduced values of diaphragm excursion and DTF could be strong indicators of extubation failure. This finding highlights that diaphragmatic ultrasound is an excellent technique for predicting weaning outcomes in infants on IMV.

This study has some limitations: first, the small sample size limits the ability to draw firm conclusions. Other limitations include the high heterogeneity of the studied population and the inability to control for other factors such as the use of medications that could influence muscle trophism, such as sedatives, neuromuscular blockers, and corticosteroid therapy. It was not possible to control the administration level of invasive mechanical ventilation, such as the pressure levels and oxygen provided to the patients.

## 6 Conclusion

Ultrasound assessment of diaphragm thickness, DTF and DE represents a promising and easily applicable tool for predicting diaphragmatic changes in preterm newborns on conventional invasive mechanical ventilation. The findings suggest that diaphragmatic deterioration during IMV is associated with poorer clinical outcomes, including prolonged hospital stays. This emphasises the importance of monitoring the diaphragm muscle using ultrasound to predict complications and optimise respiratory management in preterm newborns.

## Ethics Statement

Our investigations were carried out following the rules of the Declaration of Helsinki. This study was approved by the Research Ethics Committee (CEP) of the Santa Casa de Misericórdia do Pará Foundation, under Opinion No. 6.804.607. and the participants signed the Free and Informed Consent Form.

## Conflicts of Interest

The authors declare no conflicts of interest.
